# Female and male treatable mortality: socioeconomic and public finance related factors across European countries

**DOI:** 10.3389/fpubh.2024.1477402

**Published:** 2024-12-06

**Authors:** Aida Isabel Tavares

**Affiliations:** ^1^CEISUC – Centre for Health Studies and Research, University of Coimbra, Coimbra, Portugal; ^2^CiBB – Center for Innovative Biomedicine and Biotechnology, University of Coimbra, Coimbra, Portugal; ^3^ISEG, UL - Lisbon School of Economics and Management, University of Lisbon, Coimbra, Portugal

**Keywords:** treatable mortality, socioeconomic factors, public finance, Europe, quantile regression

## Abstract

**Background:**

About 36.5% of premature deaths in European Union countries could have been avoided through prompt and effective medical treatment. This treatable mortality is even a priority established in Sustainable Development Goal (SDG) target 3.4. Given the gap in the literature about the socioeconomic drivers of this type of mortality, as well as the increasing importance of public financial management in defining priority policies, this study aims to analyze the socioeconomic and public finance drivers associated with treatable mortality for women and men across European countries.

**Methods:**

Eurostat data is collected for 31 countries for the period 2011–2019 stratified by sex. Panel data quantile regression with fixed effects and conditional mean panel data model using feasible generalized least squares are estimated to explain treatable mortality in women and men.

**Results:**

Key findings point to a positive association between the public finance indicator proxying health priority and the treatable mortality rate for both sexes; a difference between drivers of treatable mortality between men and women; and a different set of drivers across the different quantiles of treatable mortality.

**Conclusion:**

Drivers of male and female treatable mortality may differ according to the country’s level of mortality rate. Government health priority seems to account for previous treatable mortality rates as a reactive measure. Policymakers aiming to reduce treatable mortality are likely to use instruments such as health expenditure, improved employment, education levels, and perhaps proactive policy-setting priorities concerning health.

## Introduction

The European Union (EU) witnessed premature deaths of 1,015,225 individuals under the age of 75 prior to the COVID-19 pandemic in 2019. Of these fatalities, approximately 36.5% (a total of 371,570 people) could have been prevented through prompt and effective medical treatment. The incidence of preventable death is notably higher among men, accounting for approximately 56% of cases and 44% among women.

Treatable mortality refers to deaths that could have been prevented through timely and effective healthcare interventions, including secondary prevention and treatment, following the onset of a disease, with the primary aim of reducing case fatality. The leading causes of this type of death are ischaemic heart disease, colorectal cancer, cerebrovascular disease, and breast cancer in women (1). This measure serves as a valuable indicator of health care system efficacy and as an indicator of economic development. Its importance has been magnified and given greater priority by policymakers in the implementation of Sustainable Development Goal (SDG) target 3.4. This target explicitly aims to reduce premature mortality resulting from non-communicable diseases to foster mental health and well-being, because low-quality health systems are related with high mortality rates (2). Moreover, premature mortality represents capital losses and GDP losses, thus justifying the promotion of high-quality healthcare under universal health coverage and of socioeconomic determinants of health (3).

Conceptual clarification may be necessary at this stage. Treatable mortality is a component of premature or avoidable mortality, as defined by Eurostat (4). The other component is preventable mortality, which also refers to deaths before the age of 75; however, it includes the causes of death that can be mainly avoided through effective public health and primary prevention interventions. These causes include some infectious diseases (e.g., HIV/AIDS), some forms of cancer (e.g., lung cancer), some respiratory diseases (e.g., influenza), injuries, and alcohol-and drug-related disorders (1). The difference between treatable and preventable mortality is related to the moment that healthcare intervention could have occurred. While preventable mortality refers to public health intervention before the onset of a disease or injury, treatable mortality refers to public health intervention after disease onset. Here, the focus is on treatable mortality.

**Figure 1:**
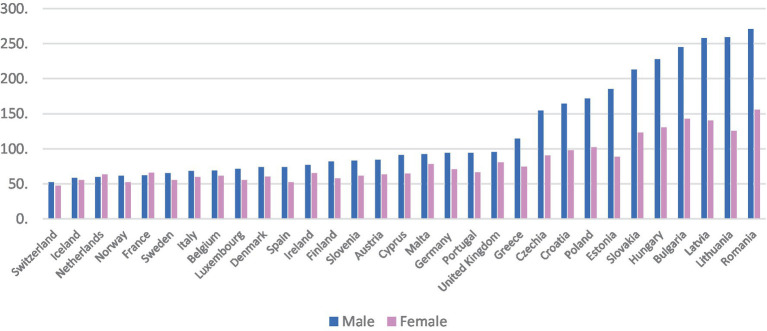
Treatable mortality in EU countries, 2019. Source: Eurostat [online code hlth_cd_apr] (21).

The objective of this study is to examine the socioeconomic and public finance drivers associated with treatable mortality for women and men across European countries for the period 2011–2019 (it does not aim to explain sex differences). To accomplish this, we estimate a fixed effects panel data linear model and quantile panel data linear regression with country controls stratified by sex for EU countries. This study addresses a gap in the existing literature about the drivers of treatable mortality, particularly public finance drivers, and contributes to understanding the underlying trends that account for variations in this health outcome indicator across European countries (Figure 1). In fact, recently at the iHEA World Congress, it was concluded that despite the strong theoretical link between public financial management and health, the empirical evidence is weak (5).

The socioeconomic determinants of health outcomes (6) have been thoroughly analyzed and discussed by several authors (7–11). An extensive review (12) lists the most important socioeconomic variables according to their statistical performance for predicting mortality, such as economic inequality, social welfare, economic performance, unemployment, and material deprivation. A small set of studies are worth mentioning because of their valuable contribution to the literature based on the estimation of panel data models (10, 13–19) (Supplementary Table S10). Although all studies include GDP *per capita* and health expenditures as independent variables, none include variables that capture the influence of public finance, specifically two indicators proposed by Kutzin (20), fiscal capacity and health priority setting with respect to the allocation of public resources. These two indicators are, respectively, proxied by the percentage of general government spending in GDP and by general government health spending (where expenditure resulting from taxes and from compulsory social insurance contributions is to be included) in general government spending. Although fiscal capacity is more subject to macroeconomic conditions on which the government may have less influence in the short-run, such as increasing tax revenues, health priority is less subject to these macro-contextual factors and more likely to be influenced by government choices in the short-run.

## Materials and methods

### Data and variables

The data utilized in this analysis was sourced from the Eurostat database (21), covering a span from 2011 to 2019, encompassing 31 European countries (Supplementary Table S1).

### Dependent variable

For this analysis, the dependent variable employed is the yearly standardized rate of treatable mortality among residents younger than 75, stratified by gender [online code ‘HLTH_CD_APR’].

### Independent variables

The explanatory variables, abbreviation, description, and Eurostat online code are shown in Table 1. This set of variables includes several macroeconomic controls, including two variables capturing the public finance features, meaning the fiscal capacity and governmental priority for health.

**Table 1:** Description of independent variables.

Variable	Abbreviation	Description	Eurostat online code
GDP *per capita*	GDPpc	Natural logarithm of purchasing power adjusted GDP *per capita*.	SDG_10_10
Public health expenditures	PHE	Government schemes and compulsory contributory health care financing schemes as percentage of GDP.	HLTH_SHA11_HF
Out-of-pocket payments	OOP	Out-of-pocket expenditure as a percentage of current health expenditure.	HLTH_SHA11_HF
Fiscal_Capacity	Fiscal_Cap	Total general government expenditure as a percentage of GDP.	GOV_10A_EXP
Priority_Health	Prior_Health	Government health expenditure as percentage of total general government expenditure.	GOV_10A_EXP
Education m/f	Education	Population by educational attainment level, Tertiary education (levels 5–8), percentage of people from 15 to 64 years.	EDAT_LFSE_03
Unemployment m/f	Unemploym	Unemployment as percentage of population in the labour force.	UNE_RT_A
Risk poverty or social exclusion m/f	Risk_Pov	Persons at risk of poverty or social exclusion percentage of population.	ILC_PEPS01
Unmet healthcare needs m/f	Unmet_HN	Self-reported unmet needs for medical examination, too expensive or too far to travel or waiting list, percentage population aged over 16 years.	HLTH_SILC_08

m, male; f, female.

### Quantitative analysis

First, we present a descriptive analysis of the variables and display the pairwise correlation between all the variables. Then we undertake some preliminary tests: (i) a Silk-Wilkson test for normal distribution of variables; (ii) a VIF estimation to check for multicollinearity; (iii) Cook’s distance to check for outliers and influential data; (iv) a Hausman test to compare fixed or random effects.

Finally, we obtain quantile plots for treatable mortality, and we estimate a quantile regression with fixed effects (22). Additionally, conditional mean panel data regression with fixed effects for women and men is estimated, as well as a panel data model using feasible generalized least squares (FGLS) to check robustness. A post-estimation Wald test is performed which allows the evaluation of the global significance of the estimated models. The analysis is performed in STATA 16.

## Results

The descriptive statistics for the panel data and pairwise correlations between variables are, respectively, presented in Supplementary Tables S2, S3. Despite some moderate level of correlations within the interval 0.5–0.7, there is no multicollinearity as displayed in Supplementary Table S4. The Cook’s distance score is below 1, indicating the absence of outliers for the male and female samples. The Shapiro–Wilk test for normal data is displayed in Supplementary Table S5. The descriptive statistics for treatable mortality rate across the whole data for males and females is presented in Table 2.

**Table 2:** Treatable mortality rate overall description statistics.

	Mean	Std. Dev.	Min	Max
Males	132.92	73.97	52.02	333.31
Females	88.15	32.88	47.54	177.74

The quantile plots can be found in Supplementary Figures S1, S2. Accordingly, it can be seen that in general female data are smooth, meaning there is no jump in data, while for male data there is a clear jump at about 70% of the fraction of treatable mortality data. For this reason, the quantiles used to estimate the quantile regression are the 30th, 50th, and 70th, used for both sexes for the sake of comparison. The statistics description by deciles of treatable mortality rate are presented in Supplementary Table S6. For instance, the 30th percentile takes the value 82.83 for males mortality rate, while for women it takes the value 66.05.

The Hausman test indicates the existence of fixed effects for the female samples but for the male sample, we may accept fixed effects with a statistical significance level of 0.0756. For this reason, we present the estimation of fixed effects and random effects for the male sample. The results for the panel data regressions based on the conditional mean are presented in Supplementary Table S7. Additionally, the estimation of the panel-data models using feasible generalized least squares is presented in Supplementary Table S8.

In general, the results show that there are more statistically significant drivers for men than for women. For both men and women, GDP, health expenditure, both public/compulsory and OOP expenditure, and education contribute to the decrease of treatable mortality. Also, for both samples, government health priority has a positive association with the treatable mortality rate, though in some instances the *p*-value is between 0.5 and 0.8. What distinguishes the results between women and men is the statistically significant role of variables related to socioeconomic inequalities. For men, unemployment tends to increase the rate of treatable mortality; on the other hand, risk of poverty (for the feasible generalized least squares estimation) and unmet health care needs have also a positive association with mortality.

Lastly, the quantile panel data regressions are presented in Table 3. Concerning these results and firstly, as expected variables GDP *per capita* and Health expenditure are statistically significant variables that contribute to reducing the mortality rate across all quantiles of the distribution. Fiscal_Cap is never statistically significant across all quantiles.

**Table 3:** Quantile panel data regressions.

	Males		Females	
**0.3 Quantile regression**	**Coef.**	***P*** > ***z***	**Coef.**	***P*** > ***z***
GDPpc	−72.761	<0.001	−48.729	<0.001
PHE	−6.710	0.001	−3.667	0.033
OOP	−0.587	0.274	−0.517	0.091
Fiscal_cap	−0.131	0.719	−0.110	0.683
Prior_Health	2.012	0.045	0.747	0.298
Education	−0.788	0.008	−0.199	0.269
Unemploym	0.623	0.063	0.204	0.382
Risk_Pov	−0.652	0.049	−0.132	0.606
Unmet_HN	−1.181	0.007	−0.361	0.134
**0.5 Quantile regression**				
GDPpc	−65.827	<0.001	−48.395	<0.001
PHE	−6.836	<0.001	−4.270	0.002
OOP	−0.743	0.114	−0.486	0.051
Fiscal_cap	−0.051	0.873	−0.029	0.894
Prior_Health	2.214	0.012	1.259	0.033
Education	−0.944	<0.001	−0.291	0.048
Unemploym	0.697	0.018	0.244	0.199
Risk_Pov	−0.500	0.086	−0.119	0.566
Unmet_HN	−1.089	0.004	−0.260	0.187
**0.7 Quantile regression**				
GDPpc	−54.249	<0.001	−47.953	<0.001
PHE	−7.047	0.006	−5.066	0.007
OOP	−1.003	0.140	−0.446	0.186
Fiscal_cap	0.083	0.858	0.078	0.793
Prior_Health	2.552	0.045	1.935	0.014
Education	−1.204	0.001	−0.413	0.037
Unemploym	0.819	0.054	0.296	0.248
Risk_Pov	−0.247	0.556	−0.103	0.714
Unmet_HN	−0.937	0.091	−0.126	0.635
Number of obs = 251				

Second, for lower levels of treatable mortality, the 30th quantile, statistically significant independent variables exist for the male sample other than GDP and PHE. However, the signs found for Risk_poverty and Unmet_HN show a negative correlation, whereas for the variable Health_priority there is a positive correlation.

At the 50th quantile of treatable mortality, the regression of females presents other statistically significant variables: Health expenditure, OOP, Health_Prior, and Education. For males, there are two additional significant variables: Unemploym and Unmet_HN, which maintain the negative correlation. For both men and women, we found a positive association between health priority and treatable mortality.

Finally, for the 70th quantile, the results for women are identical to those obtained for the 50^th^ quantile while for men the variable Unmet_HN loses statistical significance.

## Discussion

This study reveals that public health prioritization is, in general, positively associated with treatable mortality rates, whereas no evidence was found in previous studies concerning fiscal capacity. Additionally, the drivers of treatable mortality differ by gender, and these drivers vary across quantiles, indicating that factors affecting mortality rates differ depending on the level of mortality.

First, results obtained from different estimation techniques showed a positive association between the public finance indicator, that proxy health priority, and treatable mortality rate for both sexes. This is, to some extent, an unexpected correlation as one would associate higher public health priority with better health outcomes and lower treatable mortality. This could be explained by potential inefficiencies in the allocation of public funds that do not necessarily improve health outcomes. These inefficiencies could arise from mismanagement, failed targeted public health interventions, or other systematic healthcare delivery issues.

However, it could be argued that the positive correlation found here expresses the relationship between public health expenditure levels decided following the observed levels of treatable mortality. That is, a government decides on health priority for year N + 1, based on the observed treatable mortality rate in year N. Therefore, increasing levels of treatable mortality will be associated with higher levels of health priority, not only because there is a public health problem to be addressed but also for political cycle reasons. Unfortunately, we have not found another empirical study using the same public finance variables and time series available is too short to allow for a causality analysis. However, close to our finding, Berger and Messer (23) found that increasing all causes of mortality rate was associated with increasing public (excluding mandatory social contributions) expenditure as a share of total health expenditures, but no dynamic or causal analysis was performed by those authors.

Concerning fiscal capacity, we did not find any statistical evidence to support a correlation with treatable mortality. One may expect this to happen, as this indicator is better suited for long time series analysis because government’s influence over it is usually small and sluggish. On the other hand, it could also be that this indicator is not directly related with public health decisions and its effects on health outcomes. In this way, broader fiscal capacity alone seems to be insufficient to influence directly treatable mortality without efficient and targeted health expenditure (24). Although no previous study uses this indicator and relates it to some health outcome indicator, if we consider that low fiscal capacity may be related to a country’s high indebtedness, then debt results in a deterioration of health outcome indicators in the long run (25), in line with the much earlier proposal, in 1986, by Sell and Kunitz (26).

Second, findings point to a difference between the drivers of male and female treatable mortality. In general, women seem less influenced by inequality in socioeconomic factors, except for education, whereas men seem to be more susceptible to the influences of socioeconomic status such as unemployment, the risk of poverty or social exclusion, and unmet healthcare needs.

Common drivers of female and male treatable mortality are GDP *per capita* and public health expenditure. These associations were expected and are in line with the results reported in the literature (10, 13–17, 19, 27–31). Higher GDP *per capita* and higher public health expenditures are associated with better health outcomes, including lower treatable mortality. A higher GDP *per capita* reflects greater economic resources available for both individuals and governments. Larger financial resources for individuals allow them to access better living conditions and better healthcare quality; larger public health expenditures finance more investment needed to enhance healthcare infrastructure and improve healthcare interventions. Nevertheless, causal links between lower mortality and higher public health expenditures are seldom established (29), and the mechanisms and quality of public financial management are not well-understood (30).

Another common driver for women and men is education. The relationship between higher education levels and better health outcomes is well-known (32, 33). Concerning socioeconomic drivers, treatable mortality among men is associated with unemployment, and we did not find evidence that this affects women. This result is recognized elsewhere (14, 34, 35). Results also indicate a negative association between risk of poverty and treatable mortality and between unmet healthcare needs and treatable mortality. These two results are unexpected and seem to indicate the apparently wrong direction of the correlation. One expects that people affected by poverty have limited access to factors that foster good health, such as nutrition and healthy foods, shelter, safe neighborhoods in which to learn, live and work, clean air and water; thus, they would be more likely to experience illness (10, 36, 37). Our findings point in the opposite direction, which is in line with Jorgensen et al. (38) for Norway. However, a careful analysis of this result may indicate the existence of social policies to improve living conditions and access to healthcare for people at risk of poverty or social exclusion, especially in countries with higher GDP *per capita*. In fact, observing the scatter plot between male mortality and male risk of poverty (Supplementary Figure S3) and considering the pairwise correlation of −0.67, it is clear that risk of poverty is associated the higher mortality. However, when accounting for the influence of GDP *per capita*, the sign of the association changes (Supplementary Table S9), indicating the potential existence of other variables influencing the relationship between risk of poverty and treatable mortality.

An identical interpretation can be applied to the results regarding the negative association between unmet healthcare needs and treatable mortality in males. One would expect unmet health care needs would lead to poorer health outcomes and eventually to premature mortality (39). Our result is in the same direction as Lindstrom et al. (40), who found that for the age group 18–64 years there was no significant association between unmet healthcare needs and mortality. However, when controlling for GDP *per capita*, the negative effects of unmet healthcare needs are mitigated because of welfare policies in European countries are designed to lessen social inequalities. In fact, policies such as income support, and social insurance programmes contribute to reducing poverty, promoting housing access, and addressing other social determinants of health. These policies create a safety net that mitigates health disparities by fostering a more equitable distribution of health resources, and thus reducing treatable mortality rates (41).

Third, the findings show that depending on the level of mortality rate, different sets of drivers are active in women and men. Hence, no single recipe for all countries exists that aims to improve treatable mortality (42). Each country has its own sex inequality specific settings requiring specific policy measures. Nevertheless, some general trends may be established according to the level of mortality rate of each country.

For countries with lower values of treatable mortality among women, only two drivers play a role in mitigating this health outcome. These are GDP *per capita* and public health expenditures. For median or higher levels of female treatable mortality, a higher percentage of women with tertiary education also contributes to reduced mortality. Therefore, countries in Eastern Europe may use the instrument of education to improve women’s health outcomes and prevent treatable mortality (14, 16).

There are several drivers associated with male mortality in countries with lower rates of mortality, and the size of this set of drivers decreases as the level of mortality rate increases. Therefore, for median rates of male mortality, unemployment is associated with mortality, education reduces it, and unmet health care needs are negatively associated with those deaths; and for higher rates of male mortality, while education decreases mortality, unemployment increases that death rate. For countries with higher rates of mortality (such as those captured by the 70^th^ quantile and above), the only significant driver of treatable mortality that differs between men and women is unemployment, as some studies point to a larger effect of unemployment in men (43).

## Conclusion

The study presented has three key findings. The first key finding is the positive association between the public finance indicator that proxy health priority and the treatable mortality rate for both sexes, except for women, when considering the lowest rates of mortality across countries. On the other hand, the indicator of fiscal capacity seems to be mute concerning the association with treatable mortality.

The second key finding is the difference in drivers of treatable mortality between men and women. For women, treatable mortality is generally explained by GDP *per capita*, health expenditures, education, and government health priority, whereas men’s treatable mortality drivers also include socioeconomic indicators.

Finally, the last key finding concerns the different sets of drivers for treatable mortality across the different quantiles of treatable mortality; that is, statistically significant drivers differ across quantiles.

The drivers of male and female treatable mortality may differ according to the country’s level of mortality rate. Government health priority defined within public financial management is positively associated with treatable mortality; this finding may suggest that government setting priorities accounts for previous treatable mortality rates as a reactive decision. Policymakers aiming to reduce treatable mortality are likely to use instruments like health expenditures, improve employment and education levels, and be proactive in setting priorities concerning health. The potential mechanisms underlying these instruments to reduce treatable mortality rates may work through several pathways. One pathway is increasing access to primary healthcare services, preventive care, early diagnosis and treatment via government expenditure. Another pathway is improving employment opportunities, which not only broadens access to healthcare, but also leads to better labor contracts that include health insurance. Lastly, rather than relying on reactive health policies focused on hospital care, proactive policies—such as vaccination and screening programs, health literacy initiatives, the promotion of healthier lifestyles, and workplace and traffic accident prevention campaigns—activate multiple pathways to achieve the desired health outcomes.

The analysis in this study has strengths and limitations. The major strength of this work is its contribution to the literature, for which there is a gap in terms of examining the socioeconomic determinants of treatable mortality rate for men and women. Additionally, it explores the association between public finance indicators and treatable mortality, which has not been explored previously and for which comparisons with other empirical evidence are not possible. There are some limitations that should be noted. First, the sample dataset is small; specifically, it considers a short period, thus, causality cannot be concluded. The results express associations or correlations between variables. Nevertheless, the analysis does yield clear and well-founded clues for policymakers regarding the relevance of public finance to health outcomes. Future work will extend the period of time to perform a causality analysis between public finance indicators and public health outcomes. Causality analysis between health outcomes and public finance indicators, including public health expenditures, is underexplored due to the complexity of isolating the direct effects of those indicators from other influencing factors like macroeconomic conditions, individual’s lifestyles choices, and health system efficiency. Additionally, the time lag between any public expenditure and an observable health improvement, coupled with data limitations and methodological challenges, makes such analysis difficult to execute (41). Despite these difficulties, causal analysis allows for understanding how specific variables directly influence health outcomes, beyond correlation. For instance, it may allow researchers to determine whether an increase in public health expenditure directly improves treatable mortality rates or if observed improvements are due to other underlying factors such as socioeconomic conditions and healthcare delivery.

A second limitation relates to the public finance indicators used, as they do not capture the efficiency or equity of spending. Future research could address this by considering the cost-effectiveness of health expenditure by measuring changes in treatable mortality per additional monetary unit spent, and include indicators that reflect the distribution of health spending across lower socioeconomic groups to assess how well vulnerable populations are served. Nevertheless, the public finance indicators used in this investigation provide a solid measure of the overall fiscal orientation toward health and are also comparable across countries and over time.

## Data Availability

Publicly available datasets were analyzed in this study. This data can be found at: https://ec.europa.eu/eurostat/data/database.
