# Supplementary material for: Female and male treatable mortality: socioeconomic and public finance related factors across European countries
Source: Front Public Health. 2024 Dec 6;12:1477402. doi: 10.3389/fpubh.2024.1477402 (PMC11660794; doi:10.3389/fpubh.2024.1477402)
Supplement: Supplementary file 1 [file Table_1.docx]

**Supplementary Material**

Table SM1: List of countries

| Austria |  | Germany |  | Portugal |
| --- | --- | --- | --- | --- |
| Belgium |  | Greece |  | Romania |
| Bulgaria |  | Hungary |  | Slovakia |
| Croatia |  | Ireland |  | Slovenia |
| Cyprus |  | Italy |  | Spain |
| Czechia |  | Latvia |  | Sweden |
| Denmark |  | Lithuania |  | United Kingdom |
| Estonia |  | Luxembourg | | Iceland |
| Finland |  | Malta |  | Norway |
| France |  | Netherlands | | Switzerland |
|  |  | Poland |  |  |

Table SM2: Descriptive statistics

| Variable |  | Mean | Std. Dev. | Min | Max | Observations |
| --- | --- | --- | --- | --- | --- | --- |
| Treatable_m | overall | 132.92 | 73.97 | 52.02 | 333.31 | N = 276 |
|  | between |  | 74.28 | 58.75 | 293.85 | n = 31 |
|  | within |  | 9.40 | 97.43 | 172.77 | T = 8.90 |
| Treatable_f | overall | 88.15 | 32.88 | 47.54 | 177.74 | N = 276 |
|  | between |  | 32.72 | 52.05 | 164.73 | n = 31 |
|  | within |  | 5.98 | 72.83 | 107.53 | T = 8.90 |
| GDPpc | overall | 10.21 | 0.38 | 9.37 | 11.28 | N = 279 |
|  | between |  | 0.37 | 9.51 | 11.23 | n = 31 |
|  | within |  | 0.09 | 9.94 | 10.51 | T = 9 |
| PHE | overall | 6.31 | 1.82 | 2.76 | 9.83 | N = 261 |
|  | between |  | 1.79 | 3.05 | 9.37 | n = 31 |
|  | within |  | 0.32 | 5.15 | 7.81 | T-bar = 8.42 |
| OOP | overall | 21.48 | 9.51 | 8.83 | 47.74 | N = 261 |
|  | between |  | 9.62 | 9.86 | 42.32 | n = 31 |
|  | within |  | 1.32 | 11.28 | 27.07 | T-bar = 8.42 |
| Fiscal_Cap | overall | 44.66 | 7.00 | 24.30 | 62.80 | N = 270 |
|  | between |  | 6.56 | 33.26 | 56.53 | n = 30 |
|  | within |  | 2.70 | 35.53 | 58.43 | T = 9 |
| Prior_Health | overall | 13.60 | 2.90 | 5.40 | 19.70 | N = 270 |
|  | between |  | 2.86 | 6.31 | 18.36 | n = 30 |
|  | within |  | 0.71 | 10.97 | 15.89 | T = 9 |
| Education_m | overall | 24.98 | 7.18 | 11.70 | 41.30 | N = 279 |
|  | between |  | 6.99 | 13.24 | 37.86 | n = 31 |
|  | within |  | 2.05 | 17.16 | 30.47 | T = 9 |
| Unemploy_m | overall | 8.77 | 4.63 | 1.70 | 25.60 | N = 270 |
|  | between |  | 3.96 | 4.02 | 19.57 | n = 30 |
|  | within |  | 2.50 | 1.75 | 15.67 | T = 9 |
| Risk_Pov_m | overall | 22.13 | 7.26 | 10.00 | 47.70 | N = 278 |
|  | between |  | 6.92 | 12.01 | 39.72 | n = 31 |
|  | within |  | 2.54 | 12.84 | 32.16 | T = 8.97 |
| Unmet_HN_m | overall | 2.67 | 2.87 | 0.00 | 14.50 | N = 277 |
|  | between |  | 2.58 | 0.23 | 9.74 | n = 31 |
|  | within |  | 1.32 | -2.25 | 8.15 | T = 8.94 |
| Education_f | overall | 30.92 | 8.41 | 13.2 | 45.5 | N= 278 |
|  | between |  | 7.95 | 15.42 | 41.97 | n= 31 |
|  | within |  | 3.06 | 16.49 | 38.97 | T= 8.97 |
| Unemploy_f | overall | 8.91 | 5.37 | 2.4 | 31.7 | N= 270 |
|  | between |  | 4.99 | 3.74 | 27.06 | n= 30 |
|  | within |  | 2.16 | 2.31 | 13.83 | Y=9 |
| Risk_Pov_f | overall | 24.02 | 7.40 | 11.3 | 50.90 | N= 278 |
|  | between |  | 7.16 | 12.78 | 42.99 | n= 31 |
|  | within |  | 2.29 | 15.36 | 31.93 | T= 8.97 |
| Unmet_HN_f | overall | 3.58 | 3.84 | 0.0 | 17.9 | N= 277 |
|  | between |  | 3.52 | 0.267 | 13.68 | N= 31 |
|  | within |  | 1.62 | -2.34 | 10.56 | T= 8.94 |

Table SM3: Pairwise correlations

|  | Treatable_m | Treatable_f | GDPpc | PHE | OOP | Fiscal_Cap | Prior_Health | Education | Unemploym | Risk_Pov | Unmet_HN |
| --- | --- | --- | --- | --- | --- | --- | --- | --- | --- | --- | --- |
| Treatable_m | 1.000 |  |  |  |  |  |  |  |  |  |  |
| Treatable_f | 0.967 | 1.000 |  |  |  |  |  |  |  |  |  |
|  | <0.001 |  |  |  |  |  |  |  |  |  |  |
| GDPpc | -0.728 | -0.722 | 1.000 |  |  |  |  |  |  |  |  |
|  | <0.001 | <0.001 |  |  |  |  |  |  |  |  |  |
| PHE | -0.641 | -0.598 | 0.504 | 1.000 |  |  |  |  |  |  |  |
|  | <0.001 | <0.001 | <0.001 |  |  |  |  |  |  |  |  |
| OOP | 0.443 | 0.393 | -0.563 | -0.664 | 1.000 |  |  |  |  |  |  |
|  | <0.001 | <0.001 | <0.001 | <0.001 |  |  |  |  |  |  |  |
| Fiscal_Cap | -0.394 | -0.383 | 0.051 | 0.563 | -0.317 | 1.000 |  |  |  |  |  |
|  | <0.001 | <0.001 | 0.403 | <0.001 | <0.001 |  |  |  |  |  |  |
| Prior_Health | -0.202 | -0.170 | 0.267 | 0.499 | -0.612 | 0.128 | 1.000 |  |  |  |  |
|  | 0.001 | 0.005 | <0.001 | <0.001 | <0.001 | 0.035 |  |  |  |  |  |
| Education_m | -0.565 | -0.603 | 0.752 | 0.345 | -0.266 | -0.066 | -0.020 | 1.000 |  |  |  |
|  | <0.001 | <0.001 | <0.001 | <0.001 | <0.001 | 0.281 | 0.749 |  |  |  |  |
| Unemploym_m | 0.177 | 0.144 | -0.480 | -0.248 | 0.349 | 0.185 | -0.257 | -0.178 | 1.000 |  |  |
|  | 0.004 | 0.018 | <0.001 | <0.001 | <0.001 | 0.002 | <0.001 | 0.003 |  |  |  |
| Risk_Pov_m | 0.574 | 0.613 | -0.678 | -0.572 | 0.593 | -0.223 | -0.394 | -0.410 | 0.638 | 1.000 |  |
|  | <0.001 | <0.001 | <0.001 | <0.001 | <0.001 | <0.001 | <0.001 | <0.001 | <0.001 |  |  |
| Unmet_HN_m | 0.458 | 0.426 | -0.512 | -0.445 | 0.391 | -0.113 | -0.301 | -0.324 | 0.358 | 0.567 | 1.000 |
|  | <0.001 | <0.001 | <0.001 | <0.001 | <0.001 | 0.065 | <0.001 | <0.001 | <0.001 | <0.001 |  |
| Education_f |  | -0.3556 | 0.4293 | 0.0917 | -0.0601 | -0.0510 | 0.0073 | 1.000 |  |  |  |
|  |  | <0.001 | <0.001 | 0.1403 | 0.3346 | 0.4048 | 0.9046 |  |  |  |  |
| Unemploym_f |  | 0.0144 | -0.4193 | -0.2114 | 0.2958 | 0.2454 | -0.2425 | -0.1629 | 1.000 |  |  |
|  |  | 0.8143 | <0.001 | 0.0007 | <0.001 | <0.001 | 0.0001 | 0.0074 |  |  |  |
| Risk_Pov_f |  | 0.6299 | -0.6735 | -0.5936 | 0.6281 | -0.2720 | -0.4008 | -0.2711 | 0.4837 | 1.000 |  |
|  |  | <0.001 | <0.001 | <0.001 | <0.001 | <0.001 | <0.001 | <0.001 | <0.001 |  |  |
| Unmet_HN_f |  | 0.4334 | -0.5132 | -0.4549 | 0.3691 | -0.1218 | -0.2963 | -0.0207 | 0.2917 | 0.5555 | 1.000 |
|  |  | <0.001 | <0.001 | <0.001 | <0.001 | 0.0459 | <0.001 | 0.7319 | <0.001 | <0.001 |  |

Table SM4: VIF test

|  | Females |  |  | Males |  |  |
| --- | --- | --- | --- | --- | --- | --- |
| Variable | VIF | 1/VIF |  | Variable | VIF | 1/VIF |
| Risk_Pov_f | 3.12 | 0.32 |  | GDPpc | 5.07 | 0.20 |
| PHE | 3.09 | 0.32 |  | Education_m | 3.64 | 0.27 |
| OOP | 2.96 | 0.34 |  | Risk_Pov_m | 3.50 | 0.29 |
| GDPpc | 2.93 | 0.34 |  | PHE | 3.28 | 0.30 |
| Fiscal_Cap | 2.14 | 0.47 |  | OOP | 2.74 | 0.36 |
| Prior_Health | 1.84 | 0.54 |  | Unemploy_m | 2.49 | 0.40 |
| Unmet_HN_f | 1.70 | 0.59 |  | Fiscal_Cap | 2.36 | 0.42 |
| Unemploym_f | 1.68 | 0.60 |  | Prior_Health | 2.00 | 0.50 |
| Education_f | 1.43 | 0.70 |  | Unmet_HN_m | 1.57 | 0.64 |
| Mean VIF | 2.32 |  |  | Mean VIF | 2.96 |  |

Note: VIF test shows that there is no multicollinearity between independent variables, for either males or females as VIF is below 10.

Table SM5: Shapiro-Wilk W test for normal data

| Variable | Obs | W | V | z | Prob>z |
| --- | --- | --- | --- | --- | --- |
| Treatable _m | 276 | 0.818 | 36.026 | 8.380 | <0.001 |
| Treatable_f | 276 | 0.848 | 30.154 | 7.964 | <0.001 |
| GDPpc | 279 | 0.981 | 3.879 | 3.171 | 0.001 |
| PHE | 261 | 0.965 | 6.632 | 4.411 | <0.001 |
| OOP | 261 | 0.910 | 16.936 | 6.597 | <0.001 |
| Fiscal_Cap | 270 | 0.991 | 1.734 | 1.285 | 0.099 |
| Prior_Health | 270 | 0.955 | 8.699 | 5.052 | <0.001 |
| Education_m | 279 | 0.971 | 5.784 | 4.105 | <0.001 |
| Unemployment_m | 270 | 0.895 | 20.454 | 7.049 | <0.001 |
| Risk_Pov_m | 278 | 0.930 | 13.906 | 6.156 | <0.001 |
| Unmet_HN_m | 277 | 0.777 | 44.192 | 8.859 | <0.001 |
| Education_f | 278 | 0.964 | 7.189 | 4.613 | <0.001 |
| Unemployment_f | 270 | 0.812 | 36.455 | 8.398 | <0.001 |
| Risk_Pov_f | 278 | 0.933 | 13.426 | 6.074 | <0.001 |
| Unmet_HN_f | 277 | 0.790 | 41.758 | 8.726 | <0.001 |

Note: The Shapiro-Wilk test for normal data shows that none of the variables except Fiscal_Cap follow a normal distribution, which justify the use of quantile regressions.

Figure SM1: Quantile plot for male treatable mortality

Figure SM2: Quantile plot for female treatable mortality

Table SM6: Quantiles for treatable mortality rate

|  | MALES |  |  | FEMALES |  |  |
| --- | --- | --- | --- | --- | --- | --- |
| Quantile group | Quantile | Share. % |  | Quantile | Share. % |  |
| 1 | 67.64 | 4.78 | 4.78 | 58.33 | 6.26 | 6.26 |
| 2 | 75.81 | 5.47 | 10.26 | 62.24 | 6.93 | 13.19 |
| 3 | 82.83 | 5.86 | 16.12 | 66.05 | 7.12 | 20.31 |
| 4 | 90.04 | 6.57 | 22.69 | 69.72 | 7.78 | 28.09 |
| 5 | 96.74 | 6.82 | 29.51 | 74.97 | 8.05 | 36.14 |
| 6 | 107.99 | 7.77 | 37.28 | 80.19 | 8.90 | 45.04 |
| 7 | 167.77 | 9.81 | 47.09 | 97.67 | 10.00 | 55.04 |
| 8 | 216.68 | 13.72 | 60.81 | 119.18 | 11.99 | 67.02 |
| 9 | 257.97 | 18.21 | 79.01 | 145.36 | 15.56 | 82.58 |
| 10 |  | 20.99 | 100.00 |  | 17.42 | 100.00 |
| Minimum rate | 52.02 |  |  | 47.54 |  |  |
| Maximum rate | 333.31 |  |  | 177.74 |  |  |

Table SM7: Panel data linear model results

|  | MALES | RE |  | MALES | FE |  | FEMALES | FE |
| --- | --- | --- | --- | --- | --- | --- | --- | --- |
|  | Coef. | P>t |  | Coef. | P>t |  | Coef. | P>t |
| GDPpc | -72.307 | <0.001 |  | -63.711 | <0.001 |  | -48.349 | <0.001 |
| PHE | -8.013 | <0.001 |  | -6.875 | 0.001 |  | -4.352 | 0.001 |
| OOP | -0.649 | 0.044 |  | -0.790 | 0.016 |  | -0.482 | 0.022 |
| Fiscal_cap | -0.034 | 0.912 |  | -0.027 | 0.930 |  | -0.018 | 0.924 |
| Prior_Health | 2.400 | 0.005 |  | 2.276 | 0.009 |  | 1.329 | 0.015 |
| Education | -0.917 | 0.001 |  | -0.991 | <0.001 |  | -0.304 | 0.007 |
| Unemploym | 0.516 | 0.088 |  | 0.719 | 0.017 |  | 0.249 | 0.208 |
| Risk_Pov | -0.430 | 0.142 |  | -0.454 | 0.115 |  | -0.117 | 0.529 |
| Unmet_HN | -1.167 | 0.002 |  | -1.061 | 0.004 |  | -0.246 | 0.202 |
| _cons | 934.619 | <0.001 |  | 845.113 | <0.001 |  | 613.090 | <0.001 |
| N | 251 |  |  |  |  |  |  |  |
| Wald chi2(9) | 300.59 | 0.00 |  |  |  |  |  |  |
| F(9.212) |  |  |  | 31.50 | 0.000 |  | 37.27 | 0.00 |
| F test that all u_i=0: |  |  |  | F(29. 212) = 349.04 | |  | F(29.212)=124.89 | |
|  |  |  |  | Prob > F = 0.00 | |  | Prob > F = 0.00 | |
|  |  |  |  |  | |  |  | |

Table SM8: Results for Panel data linear model using GLS

|  | MALES |  |  | FEMALES |  |
| --- | --- | --- | --- | --- | --- |
|  | Coef. | P>z |  | Coef. | P>z |
| GDPpc | -64.233 | <0.001 |  | -48.180 | <0.001 |
| PHE | -5.315 | <0.001 |  | -2.903 | 0.001 |
| OOP | -0.481 | 0.086 |  | -0.442 | 0.008 |
| Fiscal_cap | -0.049 | 0.818 |  | -0.166 | 0.219 |
| Prior_Health | 1.322 | 0.050 |  | 0.806 | 0.066 |
| Education | -0.866 | <0.001 |  | -0.270 | 0.001 |
| Unemploym | 0.505 | 0.011 |  | 0.148 | 0.215 |
| Risk_Pov | -0.480 | 0.018 |  | 0.010 | 0.941 |
| Unmet_HN | -1.279 | <0.001 |  | -0.297 | 0.020 |
| _cons |  |  |  |  |  |
| Country fixed effects | yes |  |  | yes |  |
|  |  |  |  |  |  |
| Wald chi2(38) | 46,818.18 |  |  | 27,978.98 |  |
| Prob > chi2 | 0.000 |  |  | 0.000 |  |

Figure SM3: Male treatable mortality and Male risk of poverty

Table SM9: Panel data for male treatable mortality reduced model

|  | Univariate | | Multivariate | |
| --- | --- | --- | --- | --- |
|  | Coef. | P>t | Coef. | P>t |
| GDPpc |  |  | -76.762 | 0.000 |
| Risk_Pov_m | 1.789 | 0.000 | -0.341 | 0.194 |
| _cons | 93.528 | 0.000 | 924.603 | 0.000 |
| F(1.243) | 74.65 |  | 112.74 |  |
| Prob > F | 0.000 |  | 0.000 |  |
| Country fixed effects | yes |  | yes |  |

Table SM10: Selected studies based on panel data models

| Authors  (year) | Countries  (years) | Health outcomes | Independent variables |
| --- | --- | --- | --- |
| Mackenbach et al.  (2019)[10] | 15 European countries  (1990-2015) | Mortality rates and life expectancy by sex | GDPpc; health expenditure; income inequality; education; democracy index; smoking; material deprivation |
| Or  (2000)[113] | 21 OECD countries  (1970-1992) | Potential years of life lost by sex | GDPpc; total health expenditure; public health expenditure; share of white-collar employees; air pollution; alcohol, tobacco, fat, sugar consumption |
| Spijker  (2005)[14] | 43 European countries  (1968-1999) | Mortality rates by sex | GDPpc; government health expenditure; Gini index; education; share of working people in agriculture and industry; divorce rate; alcohol; air pollution; unemployment; urbanization; tobacco, fruit and cereal consumption |
| Arah et al.  (2005)[15] | 18 OECD countries  (1970-1999) | Mortality rate and potential years of life lost | GDPpc; health expenditure; tobacco, alcohol, fat, fruit and veggie, protein consumption; air pollution; physician density; doctors’ visits; share of population over 65 |
| Joumard et al.  (2008)[16] | 22 OECD countries  (1981-2003) | Life expectancy, premature mortality and infant mortality by sex | GDPpc; health spending; tobacco, alcohol, fruit consumption; education; air pollution; physician density |
|  |  |  |  |
| Roffia et al. (2023)[17] | 36 OECD countries (1999-2018) | Life expectancy at birth | GDPpc; health expenditure; out-of-pocket expenditure; physician density; hospital bed density; social spending; participation ratio to labour; prevalence of chronic respiratory diseases; temperature; total size of the population |
| Anwar et al.(2023)[18] | 8 OECD countries  (1996-2020) | Infant mortality and life expectancy | GDP; government health expenditure; number of doctors; population; CO2 emissions |
| Ivankova et al. (2022)[19] | 38 OECD countries (1994-2016) | Treatable mortality due to circulatory system diseases and endocrine, nutritional and metabolic diseases | GDPpc; health care financing |
